# Electron stream effect in 0.35 Tesla magnetic resonance image guided radiotherapy for breast cancer

**DOI:** 10.3389/fonc.2023.1147775

**Published:** 2023-07-13

**Authors:** Hsin-Hua Lee, Chun-Yen Wang, Shan-Tzu Chen, Tzu-Ying Lu, Cheng-Han Chiang, Ming-Yii Huang, Chih-Jen Huang

**Affiliations:** ^1^ Ph.D. Program in Environmental and Occupational Medicine, Kaohsiung Medical University and National Health Research Institutes, Kaohsiung, Taiwan; ^2^ Department of Radiation Oncology, Kaohsiung Medical University Hospital, Kaohsiung Medical University, Kaohsiung, Taiwan; ^3^ Department of Radiation Oncology, Faculty of Medicine, School of Medicine, College of Medicine, Kaohsiung Medical University, Kaohsiung, Taiwan; ^4^ Center for Cancer Research, Kaohsiung Medical University, Kaohsiung, Taiwan; ^5^ Department of Medical Imaging, Kaohsiung Municipal Siaogang Hospital, Kaohsiung, Taiwan; ^6^ Graduate Institute of Medicine, College of Medicine, Kaohsiung Medical University, Kaohsiung, Taiwan

**Keywords:** magnetic resonance image guided radiotherapy (MRgRT), magnetic resonance imaging (MRI), electron stream effect, breast cancer, image-guided radiotherapy (IGRT), skin dose, visibility

## Abstract

**Purpose:**

This research aimed to analyze electron stream effect (ESE) during magnetic resonance image guided radiotherapy (MRgRT) for breast cancer patients on a MR-Linac (0.35 Tesla, 6MV), with a focus on the prevention of redundant radiation exposure.

**Materials and methods:**

RANDO phantom was used with and without the breast attachment in order to represent the patients after breast conserving surgery (BCS) and those received modified radical mastectomy (MRM). The prescription dose is 40.05 Gy in fifteen fractions for whole breast irradiation (WBI) or 20 Gy single shot for partial breast irradiation (PBI). Thirteen different portals of intensity-modulated radiation therapy were created. And then we evaluated dose distribution in five areas (on the skin of the tip of the nose, the chin, the neck, the abdomen and the thyroid.) outside of the irradiated field with and without 0.35 Tesla. In addition, we added a piece of bolus with the thickness of 1cm on the skin in order to compare the ESE difference with and without a bolus. Lastly, we loaded two patients’ images for PBI comparison.

**Results:**

We found that 0.35 Tesla caused redundant doses to the skin of the chin and the neck as high as 9.79% and 5.59% of the prescription dose in the BCS RANDO model, respectively. For RANDO phantom without the breast accessory (simulating MRM), the maximal dose increase were 8.71% and 4.67% of the prescription dose to the skin of the chin and the neck, respectively. Furthermore, the bolus we added efficiently decrease the unnecessary dose caused by ESE up to 59.8%.

**Conclusion:**

We report the first physical investigation on successful avoidance of superfluous doses on a 0.35T MR-Linac for breast cancer patients. Future studies of MRgRT on the individual body shape and its association with ESE influence is warranted.

## 1 Introduction

Breast cancer has replaced lung cancer as the most frequently diagnosed cancer globally in the latest report by the International Agency for Research on Cancer (1). An estimated 685,000 women died from breast cancer in 2020, corresponding to 1 in every 6 cancer deaths in women (2). Breast cancer patients nowadays often are treated by breast-conserving surgery (BCS) followed by radiation therapy (RT). RT after BCS is indicated for ductal carcinoma *in situ* (stage 0), since RT greatly lowers the risk of local recurrence (3). In early (stage I-II) invasive breast cancer, adjuvant RT followed by BCS remains a standard of care (4). Based upon high level evidence for those with stage III–IV, RT is essential for selected patients after neoadjuvant systemic treatment followed by BCS or modified radical mastectomy (MRM) (4). Since RT may be recommended for all stages, the implications of different modalities of image guided radiotherapy (IGRT) are the keys to precision treatment for patients with breast cancer after BCS or MRM as well as for those with recurrence or distant metastasis (5).

The advance of both modern on-board imaging and planning software are required for adaptive treatment planning which had long been proposed (6). It has been challenging that thoracic radiotherapy such as that for breast irradiation has large inter-fractional and intra-fractional organ movement variation causing unwanted radiation-induced complications such as cardiac and pulmonary toxicities. Some used mechanical ventilation and surface-image mapping system to reduce the within-patient variability of breathing for breast cancer patients (7). A mounting body of evidence strongly supports IGRT (8–17). Until recently, image guidance was only performed prior to radiation treatment without simultaneous tracking. Magnetic resonance imaging-guided radiotherapy (MRgRT) has lately emerged as the state-of-the-art science in precision RT. It enables Radiation Oncologists to actually see the targets in relation to surrounding normal tissue during treatment (18, 19). Immediately after inspecting anatomical changes via MR guidance, Radiation Oncologists are able to recontour, recalculate and then execute a whole new set of treatment plan according to geographical variability at that specific treatment fraction (20–23). MRgRT offers not only novel online adaptation, but specifically better IGRT due to superior soft tissue contrast.

Up till now, IGRT in the form of MRgRT has not been prevalent. Cone beam computed tomography (CBCT) and mega-voltage CT (MVCT) remain the clinical standard for volumetric localization nowadays. It was reported that low-field MR provides better anatomic visualization of radiation targets and nearby organs at risk (OAR) as compared to CBCT or MVCT (24). Besides, MRgRT avoids redundant radiation exposure inherent to IGRT via CT (25). On the other hand, the influence from electron-stream effect (ESE) during MRgRT has been reported by few and not yet fully evaluated (26). When electrons are subjected to a magnetic field, they can be deflected from their original path, leading to a phenomenon known as the Lorentz force. Interactions between the electron beam and tissue can result in the electron air stream effect (ESE), leading to radiation being deposited outside of the intended treatment area, and the electron return effect, causing increased radiation dose to the skin and at the air/tissue interface (27). Out-of-field skin dose due to spiraling contaminant electrons in a perpendicular magnetic field has been observed (28). The data are limited for the assessment of ESE, modifiers of ESE and joint effects of radiotherapy and ESE during 0.35 Tesla MRgRT. To address these issues, we designed the current study to investigate ESE for breast cancer patients.

## 2 Materials and methods

**Figure 1:**
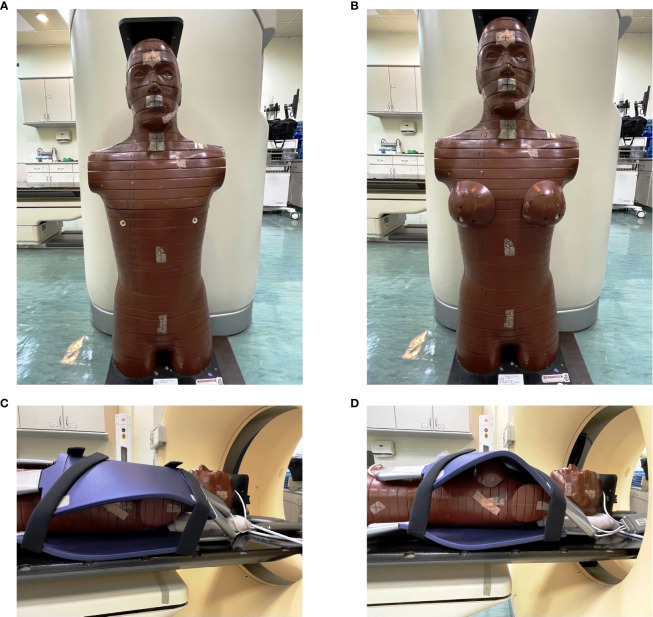
The RANDO phantom that shows **(A)** without the breast attachment in order to represent the patients after modified radical mastectomy and **(B)** with the breast attachment in order to represent the patients underwent breast conserving surgery. **(C)** The same RANDO phantom without the breast attachment that received Computed Tomography simulation with the coil on. **(D)** The same RANDO phantom with the breast attachment that received Computed Tomography simulation with the coil on.

We conducted this study on a 0.35-T MR-Linac system (MRIdian, ViewRay Inc., Mountain View, CA, USA) and used RANDO phantom to simulate the postoperative female patients with and without breast preservation (**Figure 1**). The anthropomorphic RANDO phantom conforms to the standards established by the International Commission on Radiation Units and Measurements (ICRU) Report No. 44. It was scanned with a 5-mm slice thickness using a Computed Tomography (CT) simulator (Brilliance 16 Big Bore, Philips Medical Systems, Cleveland, OH, USA). Following CT-simulation, MR-simulation was performed on MRIdian. The study was approved by the Ethical and Research Committee in the University Hospital (KMUHIRB-E(I)-20220101) and it was conducted under compliance of the Institutional Review Board regulations in accordance with the Helsinki Declaration of 1975 as revised in 1983.

### 2.1 Phantom mimicking modified radical mastectomy (MRM)

**Figure 2:**
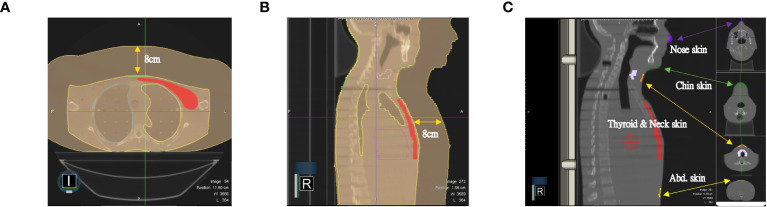
The entire structure was contoured and expanded using a 8-cm margin anteriorly and laterally to demonstrate the air as in **(A)** axial view and **(B)** sagittal view. **(C)** Five selected out-of-field locations 3 mm from the skin on the tip of the nose, the chin, the thyroid, the neck, and the abdomen.

**Figure 3:**
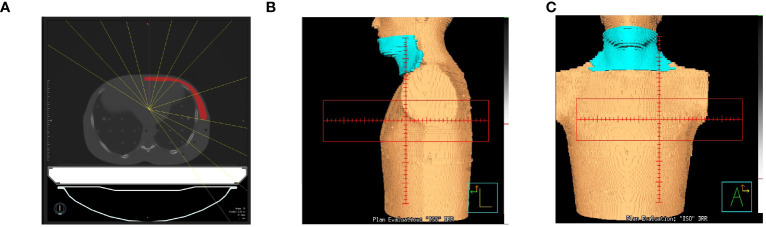
**(A)** Thirteen spaced 6-MV beams including 0°, 15°, 29°, 43°, 72°, 101°,115°, 130°, 144°, 302°, 317°, 331° and 346° were created and optimized to deliver the prescription dose with a mono-isocenter. The same angles were applied to all plans. The blue area denotes a 1cm-bolus in **(B)** the lateral view and **(C)** the front view.

As shown in **Figure 1**, we used the anthropomorphic RANDO phantom to simulate breast cancer patients after MRM for treatment planning (**Figures 1A, C**). The entire structure was contoured and expanded using a 8-cm margin anteriorly and laterally to demonstrate air with the density of 0.0012g/cm as in **Figure 2**. The external nose is a midline protuberance in the middle of the face. In this study we marked the nasal tip, the tip of external nose, which marks the termination of nasal ridge. The chin (a.k.a. the mental protuberance) lies in the midline of the mandible anteriorly. **Figure 2C** documents five selected out-of-field locations 3 mm from the surface of the tip of the nose, the chin, the thyroid, the neck, and the abdomen. We specified the skin structure as a 3 mm inner rind automatically created from the external contour (29). **Figures 3B, C** demonstrates the addition of 1cm-bolus. After all organs at risk (OAR) were contoured manually from axial CT images as described in our previous clinical study (30), we utilized the MRIdian to generate two intensity modulated radiation therapy (IMRT) treatment plans with and without bolus. The total prescribed dose was 40.05 Gy in 15 fractions. Thirteen spaced 6-MV IMRT beams were created and optimized to deliver the prescription dose with 95% PTV coverage as in **Figure 3**. The same angles with 0°, 15°, 29°, 43°, 72°, 101°, 115°, 130°, 144°, 302°, 317°, 331° and 346° were chosen with mono-isocenter and applied to all plans. **Table 1** is the constraints for OAR and planning target volume (PTV). Additionally, we use the software of ADAC Pinnacle 14.0 to make IMRT treatment plans using identical parameters in **Table 1**. There were four computerized treatment plans made for this MRM RANDO model.

**Table 1:** Constraints for planning target volume and organs at risk.

Structure	Constraints
planning target volume	V_45Gy_ ≦ 1CC;V_40.05Gy_ ≧ 95%
spinal cord	D_max_ ≦ 45 Gy
right lung	V_16.5Gy_ ≦ 950CC;V_18Gy_ ≦ 37%
left lung	V_16.5Gy_ ≦ 950CC;V_18Gy_ ≦ 37%
heart	V_42Gy_ ≦ 15CC

### 2.2 Phantom mimicking breast conserving surgery (BCS)

The anthropomorphic RANDO phantom with the breast attachments was used to simulate breast cancer patients after BCS for treatment planning (**Figures 1B, D**). The same process described in 2.1 was performed again for this model of BCS RANDO. The entire structure was contoured and expanded using a 8-cm margin anteriorly and laterally to demonstrate air with the density of 0.0012g/cm as in **Figure 2**. **Figure 2C** shows five selected locations 3 mm from the surface on the tip of the nose, the chin, the thyroid, the neck, and the abdomen. **Figures 3B, C** shows the addition of 1cm-bolus. After all organs at risks (OAR) and region of interest were delineated manually from axial CT images as described in our earlier publication (31, 32), we utilized the MRIdian to create 2 IMRT treatment plans with and without a bolus. The total prescribed dose was 40.05 Gy in 15 fractions. Thirteen spaced 6-MV IMRT beams same as those for MRM RANDO model were fashioned and optimized to deliver the prescription dose to provide 95% PTV coverage. The same angles with 0°, 15°, 29°, 43°, 72°, 101°, 115°, 130°, 144°, 302°, 317°, 331° and 346° were chosen with one mono-isocenter and applied to all plans. Additionally, we use the ADAC Pinnacle 14.0 to make IMRT treatment plans with identical parameters in **Table 1**. There were four plans produced for this BCS RANDO model.

### 2.3 Partial breast irradiation (PBI) from 2 patients’ treatment plans

Lastly, we added the images of partial breast irradiation (PBI) in two patients previously treated. Image A has right breast cancer with PVT volume of 4.4cc and image B has left breast cancer with PVT volume of 11cc. Both of them underwent BCS and received the prescribed dose of 20 Gy in one single fraction. The IMRT was performed on the Computerized Treatment Planning System of ViewRay^®^ MRIdian.

## 3 Results

### 3.1 MRM

**Figure 4:**
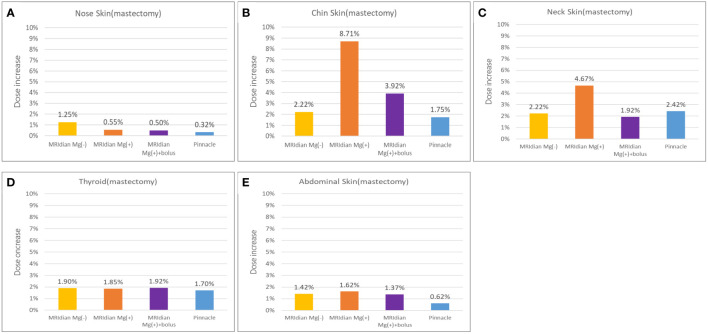
In modified radical mastectomy (MRM) model, the percentage of the redundant doses to the skin of **(A)** the nose, **(B)** the chin, **(C)** the neck, **(D)** the thyroid and **(E)** the abdomen; The dose increase in the skin of the chin **(B)** and the neck **(C)** are the most prominent in MRM RANDO model: 8.71% of the prescription dose and 4.67% of the prescription dose, respectively. When adding 1-cm bolus, the redundant dose percentages dropped from 8.71% to 3.92% and from 4.67% to 1.92% in the chin **(B)** and neck **(C)**, respectively.

**Figure 5:**
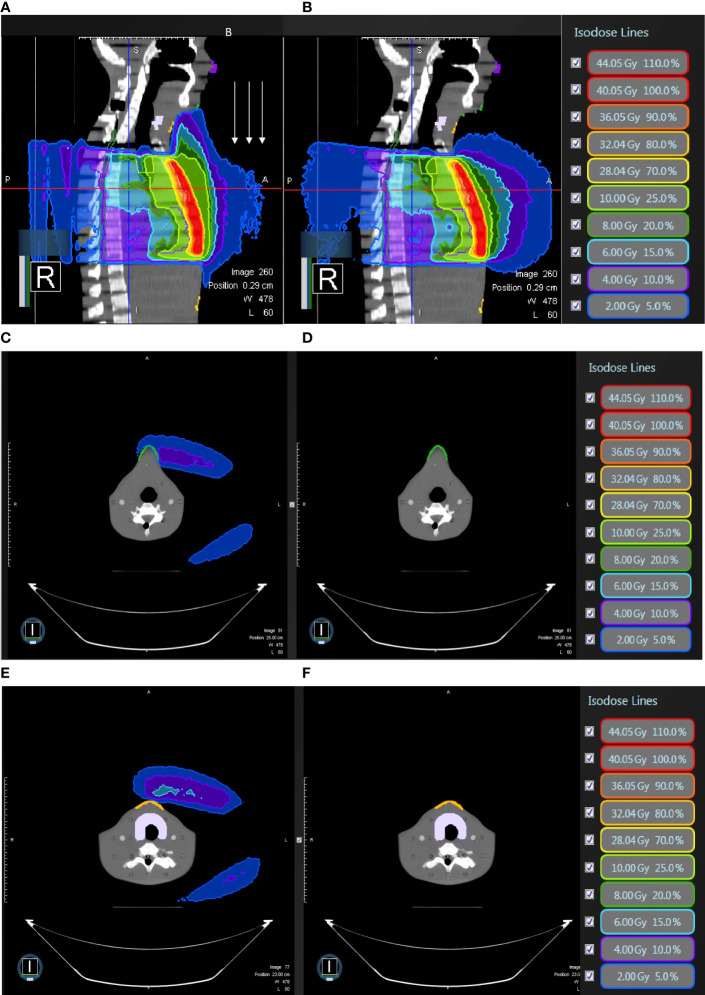
In modified radical mastectomy model, **(A)** the isodose curves under the magnetic field of 0.35T deviate toward the chin as compared to **(B)** without the magnetic field 0.35T; the dose distribution in the air near the chin with 0.35T **(C)** without 0.35T **(D)** and the neck skin with 0.35T **(E)** and without 0.35T **(F)**.

**Figure 6:**
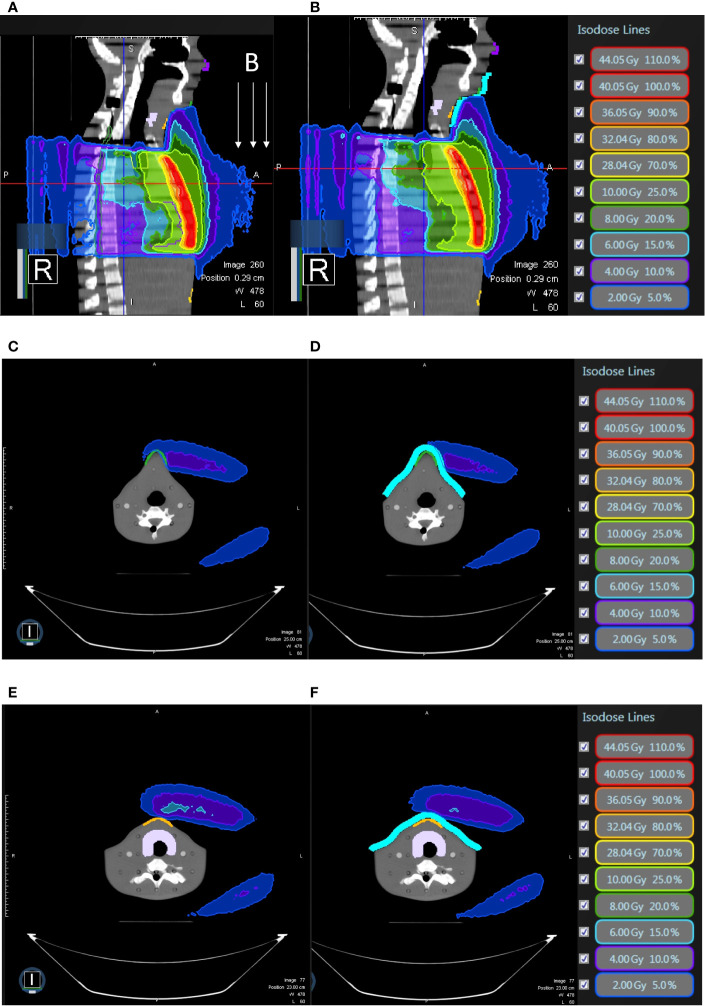
In modified radical mastectomy model, all under 0.35 T and **(A)** the isodose curves deviate toward the chin without bolus as compared to **(B)** with bolus in light blue; the dose distribution in the air near the chin without bolus **(C)** with bolus in light blue **(D)** and the neck skin without bolus **(E)** and with bolus in light blue **(F)**.

We found that the redundant dose was as high as 3.49 Gy in the skin of the chin and 1.87 Gy in the neck skin when simulating a patient with breast cancer after MRM (**Table 2**) under 0.35T with a prescribed dose of 40 Gy in 15 fractions. The maximum doses without 0.35T were 0.89 Gy for the skin of the chin and 0.89 Gy in the neck skin (both 2.22% of the prescribed dose). And the additional Pinnacle plan without 0.35T reveals 0.7 Gy for the skin of the chin and 0.97 Gy in the neck skin (1.75% and 2.42% of the prescription dose, respectively). **Figure 4** illustrates the redundant doses to the skin of chin and the neck are the most prominent in MRM RANDO model: 8.71% of the prescription dose and 4.67% of the prescription dose, respectively. **Figure 5A** shows isodose curves deviated toward the chin as compared to that without magnetic field 0.35T (**Figure 5B**). Because we had expanded 8cm out of the body surface, we were able to scrutinize the dose distribution in the air near the chin and neck (**Figures 5C-F**). The redundant doses were obviously shown in **Figure 5**. However, when we added 1-cm bolus, the redundant doses dropped 55% from 3.49 Gy in the skin of the chin to 1.57 Gy; and 58.8% from 1.87 Gy in the neck skin to 0.77Gy, respectively (**Figure 6**). The bolus effectively avoided redundant doses.

**Table 2:** Skin doses on RANDO model and the increase percentage of the presecription dose (40Gy/15fx).

	Modified radical mastectomy (MRM) / Unit: Gray															
	MRIdian 0.35T (+)				MRIdian 0.35T (-)				MRIdian 0.35T (+) + bolus				Pinnacle			
	Dmean	Dmin	Dmax		Dmean	Dmin	Dmax		Dmean	Dmin	Dmax		Dmean	Dmin	Dmax	
**Nose Skin**	0.13	0.05	0.22	0.55%	0.19	0.08	0.5	1.25%	0.12	0.06	0.2	0.50%	0.05	0.01	0.13	0.32%
**Chin Skin**	1.38	0.39	3.49	8.71%	0.56	0.28	0.89	2.22%	0.57	0.25	1.57	3.92%	0.28	0.03	0.7	1.75%
**Neck Skin**	0.71	0.41	1.87	4.67%	0.61	0.41	0.89	2.22%	0.53	0.42	0.77	1.92%	0.55	0.1	0.97	2.42%
**Abdominal Skin**	0.4	0.24	0.65	1.62%	0.38	0.18	0.57	1.42%	0.38	0.22	0.55	1.37%	0.13	0.01	0.25	0.62%
**Thyroid**	0.52	0.35	0.74	1.85%	0.53	0.34	0.76	1.90%	0.53	0.35	0.77	1.92%	0.42	0.25	0.68	1.70%

(+) with.

(-) without.

### 3.2 BCS

**Figure 7:**
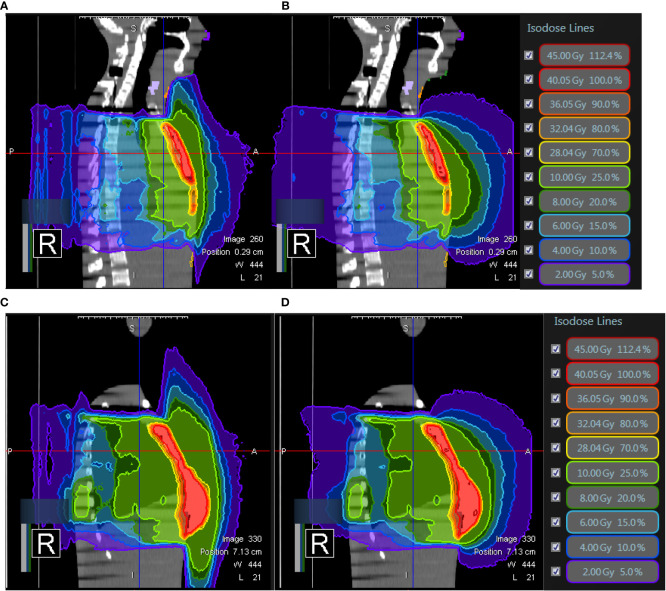
In breast-conserving surgery model, **(A)** the isodose curves under the magnetic field of 0.35T deviate toward the chin as compared to **(B)** without the magnetic field 0.35T; the dose distribution in the air in the mid-plane of the breast **(C)** with 0.35T **(D)** without 0.35T.

**Figure 8:**
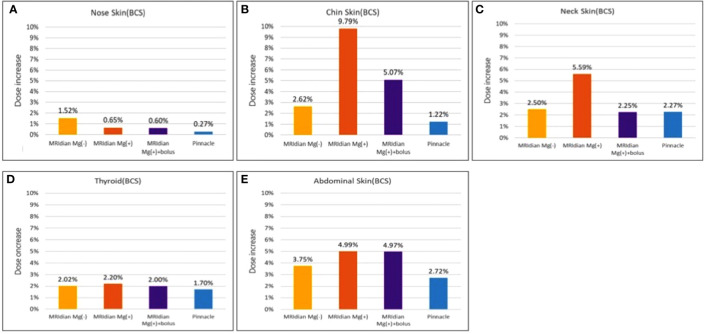
The percentage of the redundant doses to the skin of **(A)** the nose, **(B)** the chin, **(C)** the neck, **(D)** the thyroid and **(E)** the abdomen; The dose increase in the skin of the chin **(B)** and the neck **(C)** are the most prominent in breast-conserving surgery (BCS) RANDO model: 9.79% of the prescription dose and 5.59% of the prescription dose, respectively. When adding 1-cm bolus, the redundant doses dropped from 9.79% to 5.07% and from 5.59% to 2.25% in the chin **(B)** and neck **(C)**, respectively. The abdominal skin **(E)** was not affected by the bolus since the bolus covered only the chin and neck.

There was noteworthy ESE observed in the sagittal planes of the dose distribution for the simulation of patients after BCS (**Table 3**; **Figure 7**). We discovered that the redundant doses from ESE were as high as 3.92 Gy in the skin of the chin, 2.24 Gy in the neck skin and 2 Gy in the abdominal skin when simulating a patient with breast cancer after BCS under 0.35T with a prescribed dose of 40 Gy in 15 fractions. Without 0.35T, the skin doses were 1.05 Gy in the skin of the chin, 1 Gy in the neck skin and 1.5 Gy in the abdominal skin when simulating a patient with breast cancer after BCS with a prescribed dose of 40 Gy in 15 fractions. And the additional Pinnacle plan without 0.35T reveals 0.49 Gy for the skin of the chin, 0.91 Gy in the neck skin and 1.09 in the abdominal skin (1.22%, 2.27% and 2.72% of the prescription dose, respectively). **Figure 8** shows the redundant doses under the influence of magnetic field to the skin of chin, the neck and the abdominal skin are the most prominent in BCS RANDO model: 9.79% of the prescription dose, 5.59% of the prescription dose and 4.99% of the prescription dose, respectively. It was unique to note the unusual abdominal skin dose which has never been discovered in previous literatures. **Figure 7A, C** shows isodose curves deviated toward the chin as compared to that without a magnetic field of 0.35T (**Figure 7B, D**).

**Table 3:** Skin doses on RANDO model and the increase percentage of the presecription dose (40Gy/15fx).

	Breast conserving surgery (BCS) / Unit: Gray															
	MRIdian 0.35T (+)				MRIdian 0.35T (-)				MRIdian 0.35T (+) + bolus				Pinnacle			
	Dmean	Dmin	Dmax		Dmean	Dmin	Dmax		Dmean	Dmin	Dmax		Dmean	Dmin	Dmax	
**Nose Skin**	0.16	0.07	0.26	0.65%	0.26	0.09	0.61	1.52%	0.14	0.07	0.24	0.60%	0.04	0	0.11	0.27%
**Chin Skin**	1.66	0.51	3.92	9.79%	0.7	0.35	1.05	2.62%	0.71	0.31	2.03	5.07%	0.17	0.02	0.49	1.22%
**Neck Skin**	0.82	0.51	2.24	5.59%	0.73	0.56	1.00	2.50%	0.68	0.49	0.9	2.25%	0.54	0.11	0.91	2.27%
**Abdominal Skin**	1.02	0.56	2.00	4.99%	0.89	0.55	1.5	3.75%	0.99	0.57	1.99	4.97%	0.54	0.02	1.09	2.72%
**Thyroid**	0.58	0.36	0.88	2.20%	0.58	0.42	0.81	2.02%	0.57	0.35	0.8	2.00%	0.42	0.26	0.68	1.70%

(+) with.

(-) without.

**Figure 9:**
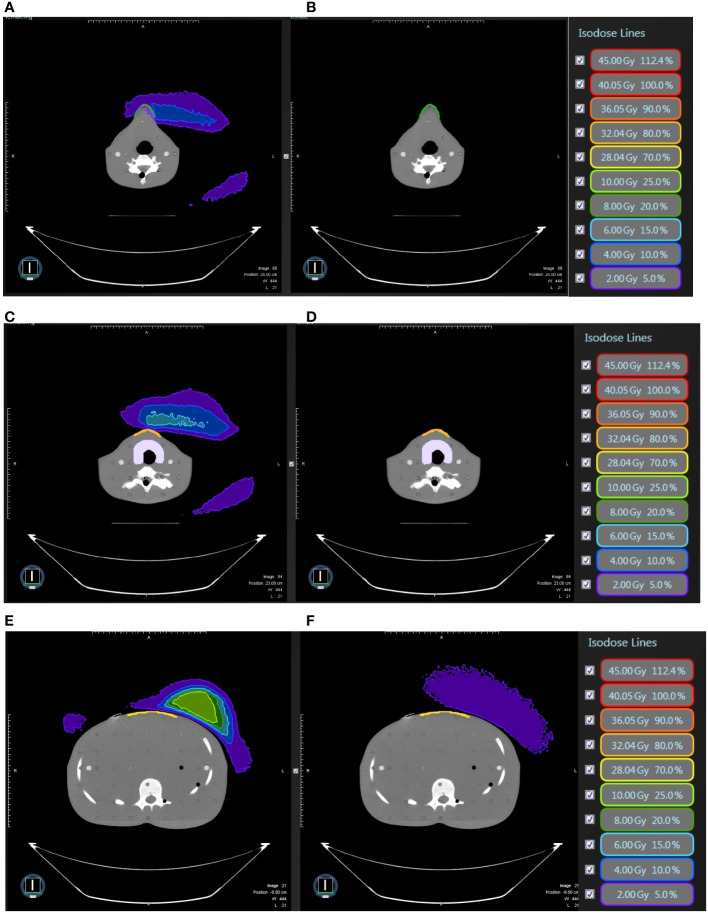
In breast-conserving surgery model, **(A)** the isodose curves deviate toward the chin as compared to **(B)** without magnetic field 0.35T; the dose distribution in the air near the neck with 0.35T **(C)** without 0.35T **(D)** and near the abdominal skin with 0.35T **(E)** and without 0.35T **(F)**. The bolus effectively avoided redundant doses.

**Figure 10:**
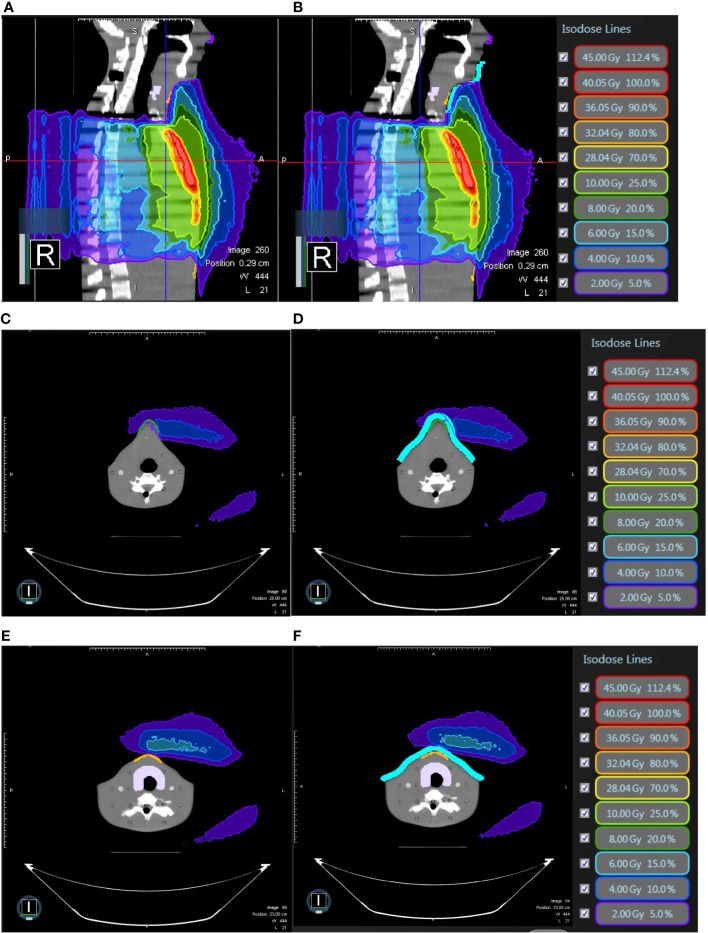
In breast-conserving surgery model, all under 0.35 T and **(A)** the isodose curves deviate toward the chin without bolus as compared to **(B)** with bolus in light blue; the dose distribution in the air near the chin without bolus **(C)** with bolus in light blue **(D)** and the neck skin without bolus **(E)** and with bolus in light blue **(F)**.

Because we had expanded 8cm out of the body surface, we were able to distinguish the dose distribution in the air near the nose, the chin, the thyroid, the neck and the abdominal skin (**Figure 9**). The redundant doses were noticeable and even greater than those of MRM RANDO model. When we added 1-cm bolus, the redundant doses dropped 48.2% and 59.8%, from 3.92 Gy in the skin of the chin and 2.24 Gy in the neck skin to 2.03Gy and 0.9 Gy, respectively (**Figure 10**). The redundant dose to abdominal skin (2Gy to 1.99Gy) was not affected by 1-cm bolus which covers only the chin and the neck (**Figures 3B, C**). Under the same condition, this demonstrates the beneficial effect of the coverage of 1-cm bolus.

### 3.3 PBI

**Figure 11:**
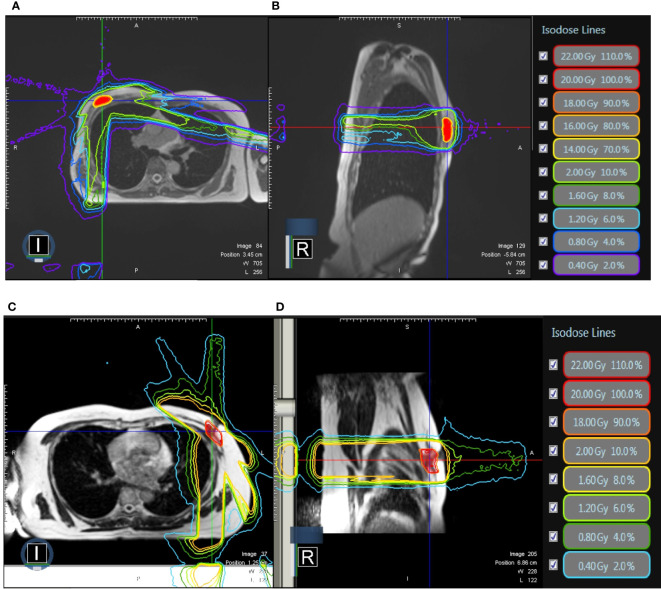
The isodose curves from two patients previously treated with a single shot of 20Gy. Both of them underwent breast-conserving surgery. **(A)** The axial view and **(B)** sagittal view of right breast cancer with a target volume of 4.4cc and **(C)** the axial view and **(D)** sagittal view of left breast cancer with a target volume of 11cc.

In the experiment of PBI (**Figure 11**), we examined the dosimetric data from two patients previously treated with a single shot of 20Gy. The plan A disclosed maximal doses of 0.03 Gy in the skin of the chin, 0.07 Gy in the neck skin, 0.18 Gy in the abdominal skin and 0.09 Gy in the thyroid under 0.35T with a prescribed dose of 20Gy in 1 fraction. The maximum doses without 0.35T were 0.03 Gy in the skin of the chin, 0.07 Gy in the neck skin, 0.13 Gy in the abdominal skin and 0.09 Gy in the thyroid. The plan B did not cover the chin and revealed maximal doses of 0.08 Gy in the neck skin, 0.22 Gy in the abdominal skin and 0.16 Gy in the thyroid under 0.35T with a prescribed dose of 20Gy in 1 fraction. The maximum doses without 0.35T were 0.1 Gy in the neck skin, 0.23 Gy in the abdominal skin and 0.16 Gy in the thyroid. There was scant difference with or without magnetic field 0.35T in both PBI plans (**Table 4**). The influence from ESE was minimal for right or left PBI.

**Table 4:** Skin doses of 2 patient plans and the increase percentage of the presecription dose (20Gy/1fx).

	Partial breast irradiation (PBI) / Unit: Gray															
	Image A								Image B							
	MRIdian 0.35T (+)				MRIdian 0.35T (-)				MRIdian 0.35T (+)				MRIdian 0.35T (-)			
	Dmean	Dmin	Dmax		Dmean	Dmin	Dmax		Dmean	Dmin	Dmax		Dmean	Dmin	Dmax	
**Chin Skin**	0.01	0	0.03	0.15%	0.02	0.01	0.03	0.15%	#	#	#	#####	#	#	#	#####
**Neck Skin**	0.05	0.02	0.07	0.35%	0.04	0.02	0.07	0.35%	0.05	0.03	0.08	0.40%	0.05	0.02	0.1	0.50%
**Abdominal Skin**	0.13	0.08	0.18	0.90%	0.1	0.07	0.13	0.65%	0.16	0.11	0.22	1.10%	0.15	0.09	0.23	1.15%
**Thyroid**	0.06	0.04	0.09	0.45%	0.06	0.04	0.09	0.45%	0.11	0.06	0.16	0.80%	0.12	0.08	0.16	0.80%

# denotes that in Image B, there was no data for skin dose on the chin because this patient has not been scanned that high to include her chin. (+) with. (-) without. #####, no data.

## 4 Discussion

To the best of our knowledge, this is the first study to access redundant doses in both WBI and PBI from 0.35-Tesla MRgRT for breast cancer patients after MRM or BCS. We not only took consideration into contemporary surgical techniques of both MRM and BCS but also modern RT strategies with both WBI and PBI. MRgRT has extended a new horizon with real-time imaging tracking which monitors intra-fractional variation. With the implementation of a combination of MRI and Linear accelerator, one may ponder is RT quality transferable. Ionizing radiation can be carcinogenic. A systematic review and meta-analysis of 762,468 patients based on European or North American populations of female breast cancer patients treated in the period between 1954 and 2007 reported that radiotherapy was associated with an increased risk of secondary non-breast cancer, especially lung cancer, esophageal cancer, and sarcoma (33). CBCT generally contributes to 0.03 Gy per scan (34). MRgRT, unlike CBCT or MVCT in IGRT, has no extra radiation dose from image guidance; it utilizes magnetic field for instantaneous imaging tracking (24). However, unnecessary doses increase because the breast shape is not parallel to the magnetic field (35). It is pressing to know how much the redundant doses out of RT field under the influence of a static magnetic field can be.

The MRLinac used in the present study consists of a split-magnet low-field (0.35 Tesla) MRI scanner with a double focused multi-leaf collimator equipped 6MV linear accelerator (36). Upgrade of the technology from ^60^Co sources to 6 MV linear accelerator improves the dose distribution and therefore reducing the low dose spread (25). Previous physics findings focused on MRLinac with ^60^Co and were insufficient for the latest model (19, 20, 37–42). This emphasizes the need for more exploration and guidelines to be incorporated into clinical decision making (43). The influence from ESE has been the latest research topic ever since the application MRgRT in clinical world (39, 44). Lately, Liu et al. has reported that the skin dose on the chin was significantly increased due ESE under 1.5 Tesla magnetic field in their study on esophageal cancer (44). It was as high as 25.2% of the prescription dose, which was even higher than that reported by Park et al., of which the corresponding maximum dose to the patient’s chin skin surface was 16.1% (39).

The purpose of the current research is to analyze ESE during RT for breast cancer patients on a MR Linac (0.35Tesla, 6MV). We discovered 9.79%, 5.59% and 4.99% of the prescription dose in the chin, neck and abdominal skin of the anthropomorphic phantom with breast attachment which was used to simulate breast cancer patients after BCS. On the other hand, 8.71% and 4.67% of the prescription dose in the chin and neck was calculated on the MRM anthropomorphic phantom model. Ten years ago, van Heijst et al. reported a pioneer study on skin dose at 0.35T and found induced effects for WBI with 2 portals or with 7 portals (35). Relative to the situation without magnetic field, the mean skin dose in WBI-2 increased by 9.5% and 12.5% at 0.35 T and 1.5 T, respectively. Although the mean skin dose in WBI with 7 portals was lower than that in WBI-2 (with 2 portals), it increased 8.2% and 6.8% at 0.35 T and 1.5 T, respectively. Though they did not investigate the effect on patients with breast cancer after MRM, they also explored PBI and concluded that the impact of the electron return effect on the skin dose is less prominent in PBI than that in WBI (35). Such finding was also noted in our present study (**Table 4**). In our daily practice on MRLinac, our medical physicists often employ 12 portals or so for optimization. In the present study, we utilized 13 portals with 0°, 15°, 29°, 43°, 72°, 101°, 115°, 130°, 144°, 302°, 317°, 331° and 346° for the best result of IMRT and still the ESE was marked.

Our current study explored the redundant doses under 0.35T and probed into the prevention measures such as the use of bolus. In our present study, the redundant doses dropped 55% from 3.49 Gy in the skin of the chin to 1.57 Gy; and 58.8% from 1.87 Gy in the neck skin to 0.77Gy, respectively for MRM model with 1-cm bolus. These would be considered unnecessary and not in alignment with ALARA principles. Or for example, this would not be a technology one would want to use in a Li-Fraumeni patient. When we added 1-cm bolus, the redundant doses dropped 48.2% and 59.8%, from 3.92 Gy in the skin of the chin and 2.24 Gy in the neck skin to 2.03Gy and 0.9 Gy, respectively in the BCS model. A recent radiomics study used gradient boosting decision tree and found that SKIN_30Gy is one of the most important factors to predict radiation-induced dermatitis higher than grade 1 (45). Another study reported the volume of skin receiving a dose >35 Gy (SKIN_V35) to be one of the most significant dosimetric predictors associated with >50% probability of radiation-induced dermatitis 2+ toxicity (46). A study working on models for normal tissue complication probability reported that on multivariate analysis, the most predictive model of acute radiation-induced skin toxicity severity was a two-variable model including the skin receiving ≥30 Gy and psoriasis [Rs = 0.32, AUC = 0.84, p < 0.001] (47). Though the skin dose observed in the present study were relatively small, optimal MRgRT should be tailored according to diverse body shapes in each individual in order to reach precision medicine. The role of post-operative radiotherapy has been strengthened by the overall survival benefit seen in breast cancer patients (4, 5). There is unmet and urgent need to improve current treatment outcomes.

MRgRT is the new quantum leap in radiation oncology. Many researchers have found that there are significant associations between unnecessary doses during radiotherapy and cardiac toxicity (10, 48). But the new concern from MRgRT may be the ESE generated with the existence of a magnetic field that work together to increase the unwanted dose (49). Our team proposes taking ESE into consideration in the assessment of clinical relevant complications including skin toxicity. Our previous studies demonstrated that IGRT improved acute skin toxicity with good long-term survival (32). The ultimate goal of this approach is to utilize IGRT in the most sophisticated form, namely, MRgRT, to provide more effective treatment strategies (49–51). We will design clinical trials from this aspect.

The drawbacks of this study include anthropomorphic phantoms limited to a single reference size, which may not be representative of the patient population of various body morphologies. This is a common downside of almost all dosimetric studies. Secondly, our work set out to create a method that could be used to avoid redundant doses from ESE, using 1-cm bolus has accomplished this partially, and not completely. Individually tailored radiotherapy in order to enhance accuracy and safety will minimize unintended exposures and low doses to peripheral organs. In the future, we aim to investigate the ESE effects of diverse patient sizes for better protection.

## 5 Conclusion

Our simulation study suggests that redundant doses from ESE during 0.35T MRgRT was more prominent in WBI for the BCS model than that in the MRM model. Besides, ESE has minimal effect on PBI. The areas greatly under influence of 0.35T MRgRT for WBI include the chin, neck and the abdomen. Bolus with the thickness of 1cm covering the chin and neck can diminish 48.2% to 59.8% of the prescription dose. For the long term goal of breast cancer treatment, extending survival and setting our sights on a cancer-free life is imperative. In order to achieve the greatest benefit from MRgRT, doses to normal tissues in or out of field must be minimized. With the effect of ESE in mind, the workflows regarding dosimetry and medical physics will be optimized from installation and throughout the lifetime of this new technology.

## Data availability statement

The original contributions presented in the study are included in the article/supplementary material. Further inquiries can be directed to the corresponding author.

## Ethics statement

The studies involving human participants were reviewed and approved by Kaohsiung Medical University Hospital. The patients/participants provided their written informed consent to participate in this study.

## Author contributions

H-HL and C-YW wrote the first draft of the manuscript, made the table, and contributed to image review and figure legends. S-TC, T-YL, and C-HC participated in treatment planning, dose calculation, and quality assurance. M-YH and C-JH conceived the methodology and supervised the experiment and revised the manuscript. All authors contributed to the article and approved the submitted version.
